# Genome of the parasitoid wasp *Dinocampus coccinellae* reveals extensive duplications, accelerated evolution, and independent origins of thelytokous parthenogeny and solitary behavior

**DOI:** 10.1093/g3journal/jkac001

**Published:** 2022-01-06

**Authors:** Arun Sethuraman, Alicia Tovar, Walker Welch, Ryan Dettmers, Camila Arce, Timothy Skaggs, Alexander Rothenberg, Roxane Saisho, Bryce Summerhays, Ryan Cartmill, Christy Grenier, Yumary Vasquez, Hannah Vansant, John Obrycki

**Affiliations:** 1 Department of Biological Sciences, California State University San Marcos, San Marcos, CA 92096, USA; 2 Department of Biology, San Diego State University, San Diego, CA 92182, USA; 3 Department of Life and Environmental Systems, University of California Merced, Merced, CA 95343, USA; 4 Department of Entomology, University of Kentucky, Lexington, KY 40506, USA

**Keywords:** parthenogenesis, Braconid wasps, phylogenomics, ancestral state reconstruction

## Abstract

*Dinocampus coccinellae* (Hymenoptera: Braconidae) is a generalist parasitoid wasp that parasitizes >50 species of predatory lady beetles (Coleoptera: Coccinellidae), with thelytokous parthenogeny as its primary mode of reproduction. Here, we present the first high-quality genome of *D. coccinellae* using a combination of short- and long-read sequencing technologies, followed by assembly and scaffolding of chromosomal segments using Chicago + HiC technologies. We also present a first-pass ab initio and a reference-based genome annotation and resolve timings of divergence and evolution of (1) solitary behavior vs eusociality, (2) arrhenotokous vs thelytokous parthenogenesis, and (3) rates of gene loss and gain among Hymenopteran lineages. Our study finds (1) at least 2 independent origins of eusociality and solitary behavior among Hymenoptera, (2) 2 independent origins of thelytokous parthenogenesis from ancestral arrhenotoky, and (3) accelerated rates of gene duplications, loss, and gain along the lineages leading to *D. coccinellae*. Our work both affirms the ancient divergence of Braconid wasps from ancestral Hymenopterans and accelerated rates of evolution in response to adaptations to novel hosts, including polyDNA viral coevolution.

## Introduction

Hymenopterans are an iconic group among the diverse and species rich insect orders and encompass expansive species radiations across sawflies, ants, bees, and wasps, dating back to the Carboniferous and Triassic periods 329–239 million years ago (MYA) (Gauld 1988; Malm and Nyman 2015; Peters *et al.* 2017; Branstetter *et al.* 2018). Current consensus across contemporary studies into resolving the phylogeny of Hymenoptera establish the divergence of sawfly and woodwasp lineages (“Symphyta”) from all other hymenopterans (“Apocrita”) near the basal branches in this order’s evolutionary history, while Apocrita further radiated into a diverse range of parasitoids (“Parasitica” clade) and stinging insects (“Aculeata” clade) which comprises stinging wasps, bees, and ants (Gauld 1988; Vilhelmsen and Turrisi 2011; Malm and Nyman 2015; Branstetter *et al.* 2018). Amongst the many evolutionary novelties to arise among Hymenopterans are differential modes of reproduction (e.g. sexual, thelytokous, and arrhenotokous parthenogenesis), ecto- and endo-parasitism, and eusocial behavior. Recent work suggests that the most recent common ancestor of Hymenoptera was phytophagous and originally consumed living plant tissues (Peters *et al.* 2017). Therefore, the transition from phytophagy to parasitism has been hypothesized to radiate from a “single endophylytic parasitoid” wasp predecessor between 289 and 211 MYA in the Permian or Triassic period (Peters *et al.* 2017). Specifically, among Braconid wasps, the Euphorinae subfamily predominantly exploits the adult stage of their hosts (koinobiosis), which is not the most common mode of host resource exploitation relative to the majority of parasitoid wasps (Stigenberg *et al.* 2015). It has also been posited that ancestral members of the Euphorine clade may have shifted host-resource exploitation from ovipositing within juvenile hosts to adult hosts which were in the same location, allowing for further adaptive radiations to their host (Quicke and van Achterberg 1990).

*Dinocampus coccinellae* (Hymenoptera: Braconidae-Euphorinae) is a parthenogenetic, generalist parasitoid wasp with a cosmopolitan distribution, observed to parasitize over 50 species of lady beetles (Coleoptera: Coccinellidae; Ceryngier *et al.* 2018) across the world. Characteristic of other Braconid wasps, parasitoid larvae feed on their insect hosts throughout development until they eclose as an adult female ready to oviposit unfertilized eggs into a host (Shaw and Huddleston 1991). However, unlike other endoparasitoids in the Euphorinae subfamily that are largely koinobionts with characteristically narrow host ranges (Shaw and Huddleston 1991), *D. coccinellae* are generalist endoparasitoids that parasitize incognito (endophytic) hosts (Ceryngier *et al.* 2018). *Dinocampuscoccinellae* is observed to predominantly reproduce parthenogenetically (thelytokous, i.e. emergence of diploid females from unfertilized eggs), with the rare occurrence of observed males in the population (Wright 1979). Little is known about their evolutionary history, or host-shifting tactics, with some recent work indicating considerable phenotypic plasticity in size-morphology of emergent daughter wasps (Vansant *et al.* 2019) covarying with the size of their hosts. Mutations and chromosomal segregation should therefore account for all genetic variation in each new generation of mostly clonal *D. coccinellae* (Slobodchikoff and Daly 1971), with no recombination.

*Dinocampus* *coccinellae* are also solitary wasps, with limited interactions between other conspecific individuals, unlike eusocial wasps. Eusociality and solitary behavior have been long proposed to have independently evolved among Hymenopteran lineages (Hines *et al.* 2007; Kuhn *et al.* 2020). Eusociality was previously interpreted to have singular origins in vespid wasps, deriving eusocial behavior from a singular common ancestor in Hymenoptera (Rehan and Toth 2015). However, through multigene phylogeny analyses, it has been observed that eusociality may have evolved twice in vespid wasps (Hines *et al.* 2007). Nonetheless, the origins of solitary behavior in Hymenoptera are yet to be delineated, primarily owing to the absence of genomic data among solitary wasps. Another interesting aspect of *D. coccinellae’*s biology involves individual wasps harboring an RNA virus (*D.coccinellae* Paralysis Virus, DcPV) that replicate in the cerebral ganglia cells of their coccinellid hosts, thereby manipulating their behavior (Dheilly *et al.* 2015). This endosymbiotic parasitic relationship between *D. coccinellae* and DcPV thus suggests the independent coevolution of genes involved in antiviral response, and host behavioral manipulation, with accelerated gene family evolution among genes involved in host–parasite conflicts.

As a first attempt to address many of these questions and to decipher the evolutionary history of *D. coccinellae*, here, we sequence the first high-resolution genome of the species, followed by a first-pass annotation and phylogenomic analysis of *D. coccinellae* in the context of other Hymenopterans sequenced as part of the i5K project. Our analyses provide the foundation for future research in understanding the genomics of host shifts, behavioral manipulation, and parthenogenesis in a unique parasitoid wasp species.

## Methods

### Samples, wasp rearing

Parthenogenetic lines of female *D. coccinellae* that were collected from the field in Summer 2018 were raised on a laboratory population of *Coccinella septempunctata* from Kansas, USA (JJO personal communication), and *Hippodamia convergens* obtained from Green Thumb Nursery, San Marcos, CA, USA. Stocks of *C. septempunctata* and *H. convergens* were maintained in separate insect tents (fed on pea aphids ad libitum*—Acyrthosiphon pisum*, raised on fava bean plants—*Vicia faba*) in a greenhouse at CSUSM, San Marcos, CA, USA. Parthenogenetic lines (>20 individual wasps) were collected from exposing female *D. coccinellae* to multiple host beetles over their lifetime, and thereon flash-frozen using liquid nitrogen, and maintained at –80°C until further processing. Genomic DNA was then extracted using the Qiagen Blood & Cell Culture DNA Mini Kit (Cat. No. 13323), following the manufacturer’s protocols (27 mg of wasp tissue was ground on dry ice, and input into 2 ml of G2 buffer, followed by pelleting and resuspension in 50 µl of TE buffer). DNA quality was then assessed using a 1% agarose gel and quantified using a Qubit 2.0 Fluorometer with broad range standards (final concentration estimate of 150 ng/µl), and a Nanodrop Spectrophotometer (A260/280 = 1.84, A260/230 = 2.03, final concentration estimate of 176.60 ng/µl).

### Chicago library preparation and sequencing (Dovetail Genomics)

The protocols of Putnam *et al.* (2016) and Lieberman-Aiden *et al.* (2009) were then used to produce a Chic (R) library and a Dovetail HiC library, respectively. Briefly, ∼500 ng of quality-assessed, high-molecular weight genomic DNA was subject to chromatin reconstitution in vitro, then fixed with formaldehyde. Fixed chromatin was then digested with the DpnII (N’B), 5′ overhangs filled in with biotinylated nucleotides, and then free blunt ends were ligated. After ligation, crosslinks were reversed and the DNA purified from protein. Purified DNA was treated to remove biotin that was not internal to ligated fragments. The DNA was then sheared to ∼350 bp mean fragment size and sequencing libraries were generated using NEBNext Ultra enzymes and Illumina-compatible adapters. Biotin-containing fragments were isolated using streptavidin beads before PCR enrichment of each library. The Chicago and HiC libraries were then sequenced on an Illumina HiSeq X at Dovetail Genomics.

### PacBio library and sequencing

The manufacturer recommended protocol was used to generate a PacBio SMRTbell library (∼20 kb) for PacBio Sequel using SMRTbell Express Template Prep Kit 2.0 (PacBio, Menlo Park, CA, USA). The library was bound to polymerase using the Sequel II Binding Kit 2.0 (PacBio) and loaded onto PacBio Sequel II. Sequencing was then performed on PacBio Sequel II 8M SMRT cells at Dovetail Genomics, generating 179 Gb of raw data.

### De novo genome assembly

The long-read assembler, Wtdbg2 v.2.5 (Ruan and Li 2020), was used to assemble the genome (–genome_size–.2g –read_ty– sq –min_read_len 5000) and for assembly polishing to build a consensus. Briefly, continuous long reads were mapped to the assembly using minimap2 and mapping information was used as an input for wtpoa-cns for polishing. Blobtools v1.1.1 (Laetsch and Blaxter) was used to identify potential contamination in the assembly based on NCBI BLAST (v2.9) hits of the assembly against the NT database. A fraction of the scaffolds was identified as contaminants and was removed from the assembly. The filtered assembly was then used as an input to purge_dups v1.1.2 (Guan *et al.* 2020), and potential haplotypic duplications were removed from the assembly.

### Scaffolding with Chicago and HiC HiRise

The input de novo assembly after filtering for contaminations and duplicate haplotypes, Chicago library reads, and Dovetail HiC library reads were used as input to Dovetail’s HiRise, a software pipeline designed specifically for using proximity ligation data to scaffold genome assemblies (Putnam *et al.* 2016). An iterative analysis was then conducted, comprising the following steps: (1) Chicago library sequences were aligned to the draft input assembly using a modified SNAP read mapper (http://snap.cs.berkeley.edu), (2) the separations of Chicago read pairs mapped within draft scaffolds were analyzed by HiRise to produce a likelihood model for genomic distance between read pairs, and the model was used to identify and break putative misjoins, to score prospective joins, and to make joins above a threshold, and (3) after aligning and scaffolding the Chicago library reads, Dovetail HiC library sequences were aligned and scaffolded following the same method. The quality of these final scaffolded assemblies was assessed using N50, N90, and other genome continuity statistics, prior to additional bioinformatic analyses. Reference-free quality of the assembly was then assessed using the kmer-completeness method implemented in Merqury v.1.3 (Rhie *et al.* 2020) at an optimal kmer size of 19 for a genome size of 182 Mb. Briefly, kmers were identified from all raw paired-end reads from the HiC libraries, followed by estimation of kmer-completeness and error rates.

### Ab initio, homology-mediated gene prediction, repeat modeling and masking

AUGUSTUS v.3.3.3 (Hoff and Stanke 2019) was used to predict protein-coding genes and coding sequences on the final HiRise genome assembly, using the *Nasonia vitripennis* genome annotation as a training set (Rago *et al.* 2016). Repeat masking was also performed on the HiRise assembly using RepeatMasker v.4.0.9 (Smit *et al.* 2019). The *Drosophila melanogaster* family in the Dfam v.3.3 library was used as a reference repeat library, and all output annotations were obtained as GFF3 formatted files. We also utilized RepeatModeler v.2.0.0 (Flynn *et al.* 2020) for de novo prediction of transposable elements and repeats and combined the de novo predictions with the reference-based prediction using RepeatMasker v.4.0.9 to obtain a comprehensive repeat library.

Additionally, we used the homology-mediated gene prediction tool GeMoMa v1.7.1 (Keilwagen *et al.* 2019) with the *Microplitis demolitor* and *Aphidius ervi* genomes and annotations obtained from InsectBase v.2.0 (Yin *et al.* 2016) as a reference to obtain GFF3 annotation tracks to supplement the ab initio predictions.

### Ortholog identification, core gene completeness

All amino acid sequences predicted by AUGUSTUS were then uploaded to OrthoDB v.10.1 and orthologous amino acid sequences were identified using 5 Hymenopteran genomes—*M.demolitor*, genome GCF_000572035.2, *N.vitripennis*, genome GCF_000002325.3, *Neodiprion lecontei*, genome GCF_001263575.1, *Orussus abietinus*, genome GCF_000612105.2, and *Trichogramma pretiosum*, genome GCF_000599845.2. Completeness of the HiRise assembly was assessed using BUSCO v.5.0 (Seppey *et al.* 2019), against core genes from all Eukaryotes (eukaryota_odb10.2019-11-20—255 BUSCO markers), Insects (insecta_odb10.2019-11-20—1,367 BUSCO markers), and Hymenoptera (hymenoptera_odb10.2019-11-20—5,991 BUSCO markers).

### Multiple sequence alignment, species tree reconstruction

A BLAST database was then constructed using the AUGUSTUS predicted gene-set, and the complete list of identified orthologs for *D. coccinellae* was then “intersected” with the list of single-copy amino acid sequences from the i5K project (Thomas *et al.* 2020) by using BLASTP and obtaining the scaffold coordinates across the *D. coccinellae* genome. Separate FASTA files (for each orthologous single-copy gene) were then constructed with all the i5K Hymenopteran genomes and our *D. coccinellae* genome, and multiple sequence alignments constructed using pasta v.1.8.6 (Mirarab *et al.* 2015). RAxML v.8.2.12 (Stamatakis 2014) was then used to construct gene trees using the PROTGAMMAJTTF amino acid substitution model, *sensu*Thomas *et al.* (2020). ASTRAL v. 5.7.7 (Zhang *et al.* 2018) was then utilized to infer an unrooted species tree.

### Time calibration, ancestral state reconstruction

The species tree obtained from ASTRAL was then time-calibrated using the fossil-times derived from Thomas *et al.* [2020; common ancestor of *Athalia rosae* and all other hymenopterans—226.4–411 MYA, common ancestor of Formicidae (ants), and Anthophila (bees)—89.9–93.9 MYA, common ancestor of Apis (honeybees) and Bombus (bumblebees), Melipona (stingless bees)—23–28.4 MYA]. Ninety-five random orthologous amino acid locus alignments were concatenated from across the 26 species analyzed (with *Zootermopsis nevadenisis* as outgroup) and analyzed using the approximate likelihood method implemented in mcmctree (Yang 2007). Briefly, the estimation of divergence times and branch lengths is conducted in 2 steps: (1) branch lengths are estimated using a maximum likelihood method and (2) divergence times are then estimated using an MCMC method. The root-age was set to be <1,000 MYA, and likelihood estimation was performed using the JC69 model, followed by a long MCMC run (2e7 iterations discarded as burn-in, followed by 1e7 iterations, sampling every 10 iterations, generating a total of 1e6 samples). Convergence of the MCMC was then assessed using Tracer 1.7.1 (Rambaut *et al.* 2018) by observing the traces of all divergence time parameter estimates, and Effective Sample Size (ESS) values. The time-calibrated rooted tree obtained from mcmctree was then used for ancestral state reconstruction using the phytools package in R (Revell 2012). Specifically, we used the (1) discrete state reconstruction and (2) empirical Bayes reconstruction using 1,000 simulated trees for 2 relevant Hymenopteran traits—(1) mode of reproduction—thelytoky (unfertilized eggs developing into females), arrhenotoky (unfertilized eggs developing into males), and sexual reproduction, (2) sociality—solitary, eusociality, and facultative sociality.

### Gene family evolution

All protein-coding gene sequences from Hymenoptera from the study of Thomas *et al.* (2020) were obtained from www.arthrofam.org and together with the ab initio protein predictions from our AUGUSTUS run, were parsed through the OrthoFinder pipeline (Emms and Kelley 2019) to perform comparative genomic analyses of (1) gene duplications, (2) identifying single-copy orthologs, and (3) delineating orthogroups based on reciprocal DendroBLAST/DIAMOND searches and estimating gene trees. The gene family counts identified by OrthoFinder and a rooted, binary, and ultrametric species tree (based on the species tree inferred above) were then used in iterative runs of the likelihood-based method, CAFE5 (Mendes *et al.* 2021) to estimate gene turnover rates (λ) and annotation error rates (ε), sensu the methods of Thomas *et al.* (2020). Additionally, a parsimony method (DupliPHY v.1.0—Ames and Lovell 2015) was used to obtain accurate ancestral gene counts. Significant rapid evolution (gene gain or loss) was then assessed by regressing gene counts at internal nodes (ancestral) vs external (extant) nodes, with statistical significance assessed at >2 standard deviations of the variance within the gene family.

## Results

### Genome assembly quality and completeness

The final HiRise assembly from Dovetail Genomics suggests an approximate genome size of 182 Mbp in 720 scaffolds, with a total of 183 gaps, GC content of 38.5%, an N50 score of 8.6 Mbp, and N90 score of 536 Kbp (Table 1, Supplementary Table 2). The largest scaffold was 19 Mbp, with 99.72% of scaffolds >1 Kbp in length, and contained an average of 10.05 missing base-calls (N’s) per 100 kbp. A majority (∼160 Mbp) of the genome was captured in the 20 longest contigs (Supplementary Fig. 2). Based on a kmer size of 19, reference-free genome completeness analyses using merqury v.1.3 estimated the assembly to be 90.75% complete, with an error rate of 0.69% and a QV (consensus quality) of 21.57. Our assembly of *D. coccinellae* is thus by far the most complete, and contiguous of all publicly available parasitoid wasp genomes in the i5K project (http://i5k.github.io/arthropod_genomes_at_ncbi). Analyses of BUSCO completeness using the eukaryota_odb10 database obtained 94.9% completeness (242 out of 255 groups searched), with >89% completeness upon comparison with the insecta_odb10 and hymenoptera_odb10 databases (Table 2). Identification of de novo repeats and transposable elements with RepeatModeler + RepeatMasker with identified a total of 1,024 retroelements (∼1.24 mbp), 2,651 DNA transposons (∼1 mbp), and other simple repeat and satellite regions (see Supplementary Table 3). A comprehensive annotation of repeats thus identified 17.38% (∼32 Mbp) of the genome to be comprised of repeats.

**Table 1.** Genome contiguity statistics from across Braconid wasp genomes that are publicly available, in comparison with the high-quality *D. coccinellae* genome of this study.

Species	Source	Genome size (Mbp)	Contig N50 (bp)	Scaffold N50 (bp)	% GC
*Dinocampus coccinellae*	This study	182.09	8,604,445	8,604,445	38.77
*Cotesia vestalis*	i5k	186.1	46,055	46,055	30.6
*Diachasma alloeum*	i5k	384.37	45,480	657,001	38.9
*Fopius arisanus*	i5k	153.63	51,867	978,588	39.4
*Macrocentrus cingulum*	i5k	127.92	65,089	65,089	35.6
*Microplitis demolitor*	i5k	241.19	14,116	1,139,389	33.1

Of note are the comparable genome sizes (126.92–384.37 Mbp), and % GC content (30.6–39.4%). Our *D. coccinellae* assembly, however, presents a several-fold improvement in contig and scaffold N50.

**Table 2.** BUSCO genome completeness measures of the *D. coccinellae* HiRise assembly, when compared to 3 separate databases: (1) eukaryota, (2) insecta, and (3) hymenoptera.

BUSCO dataset	Total searched	Complete BUSCOs	Complete, single-copy BUSCOs	Complete, duplicated BUSCOs	Fragmented BUSCOs	Missing BUSCOs
**eukaryota_odb10**	255	237 (92.94%)	227 (89.02%)	10 (3.92%)	5 (1.96%)	13 (5.09%)
**insecta_odb10**	1,367	1,311 (95.9%)	1,286 (94.1%)	25 (1.8%)	9 (0.7%)	47 (3.4%)
**hymenoptera_odb10**	5,991	5,352 (89.%)	5,290 (88.3%)	62 (1.0%)	151 (2.5%)	488 (8.2%)

BUSCO completeness assesses the quality of a genome assembly by mapping core genes and gene families that are highly conserved across taxa.

### Genome annotation and orthology

Ab initio gene prediction using AUGUSTUS v.3.3.3 (Hoff and Stanke 2019) identified a total of 68,797 protein-coding sequences in the HiRise assembly. Homology-mediated gene prediction using GeMoMa v.1.7.1 predicted combined evidence from the *M.demolitor* and *A.ervi* genomes for 23,448 protein-coding genes. All gene annotations were then added as a separate track to create a genome browser (JBrowse) instance, which can be accessed at https://usegalaxy.org/u/rykamae/h/dcoccinellaegenome

Orthology prediction using OrthoDB v.10.0 against 5 other parasitoid wasp genomes obtained over 8,000 orthogroups (longest—EGF-like calcium-binding domain 5at7399, and Immunoglobulins 0at7399). Annotations for these orthologs were obtained and corresponding GO terms associated were cataloged.

### Phylogeny, time calibration, and ancestral state reconstruction

**Fig. 1.**
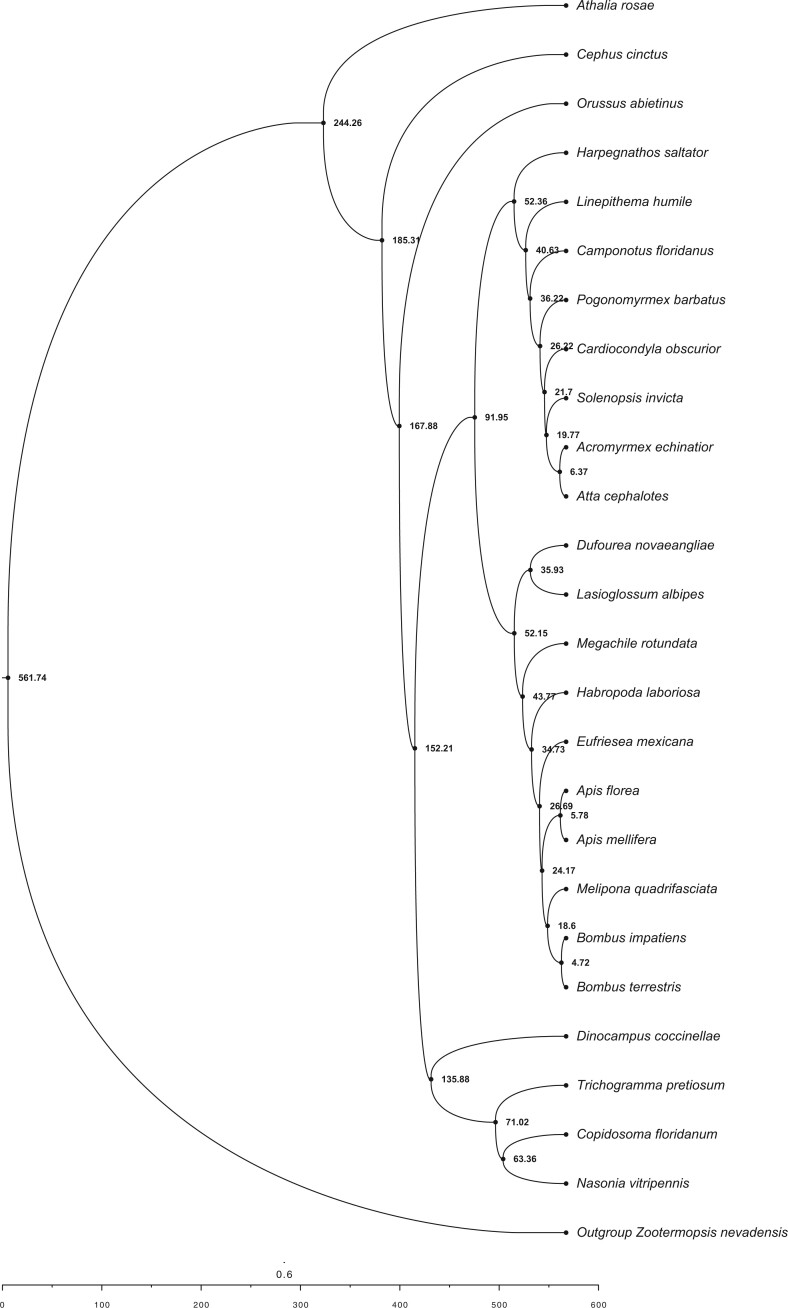
Fossil-time calibrated species tree using 200 random genes (from 2,045 total genes) across all publicly available hymenopteran genomes in the i5k Project, with *D. coccinellae* placed as being sister to other Braconid wasps. All nodes are in units of MYA, and branch lengths are scaled by time, as indicated by the scale below.

Phylogeny reconstruction of the species tree using ASTRAL from 2,045 gene trees placed *D. coccinellae* as sister to other parasitoid wasps (*T.pretiosum*, *Copidosoma floridanum*, and *Nasonia vitripensis*). The remainder of the tree replicated the same species tree topology obtained from ASTRAL and RAxML analyses from Thomas *et al.* (2020), which resolves wasps as sister to the common ancestor of all ants and bees (Fig. 1).

Fossil-based time calibration of the ASTRAL species tree obtained above with MCMCtree, and utilizing 200 randomly sampled genes (out of 2,045) determined the split of the outgroup (*Zootermopsis nevadensis*) and all hymenopterans at 561 MYA (95% HPD interval of 256.93 MYA—954.48 MYA), the split of *D. coccinellae* from other wasps (*T. pretiosum*, *C. floridanum*, *N. vitripennis*) at 135.88 MYA (95% HPD interval of 108.87 MYA—166.69 MYA), and the split of Apidae (bees) and Formicidae (ants) at 91.94 MYA (95% HPD interval of 89.92 MYA—93.91 MYA). Ancestral state reconstruction of mode of reproduction using phytools with the MCMC method of Huelsenbeck *et al.* (2003; stochastic character mapping) revealed independent evolution of thelytoky along the branches leading to *D. coccinellae* and *T. pretiosum*, with their common ancestors determined to have been arrhenotokous. Similarly, sexual reproduction was determined to have evolved independently in the common ancestor of *Attacephalotes* and *Acromyrmexechinatior.* A similar estimation of ancestral state reconstruction for sociality estimated the independent convergent evolution of eusociality within the Hymenoptera along at least 3 lineages: (1) within Apidae, in the common ancestor of *Bombusterrestris*, *Bombusimpatiens*, *Meliponaquadrifasciata*, *Apismellifera*, and *A. florea*, (2) in the common ancestor of all Formicidae, and (3) along the branch leading to *C. floridanum*. Interestingly, the common ancestor of all bees, ants, and wasps was determined to have exhibited predominantly solitary behavior, with facultative sociality evolving independently along the *Eragrostismexicana* lineage.

### Gene family evolution

Discovery of orthologous sequences using OrthoFinder with 25 Hymenoptera genomes (24 from i5k, and *D. coccinellae*) determined 96.1% of all genes analyzed assigned to 19,210 unique orthogroups, 3,116 of which contained all species, and 1,241 contained single-copy orthogroups. Interestingly, *D. coccinellae* was determined to have a total of 40,012 gene duplication events, several folds larger than other hymenopterans (nearly 10-fold higher than *N. vitripennis*, with 4,180 gene duplication events). CAFE5 estimated gene turnover rate (λ) of 0.145 (with a maximum possible λ of 0.41). DupliPHY analyses to determine accelerated rates of gene loss or gain across all single-copy ortholog families analyzed determined several significant gene loss events along the *D. coccinellae* lineage (see Supplementary Table 1) in comparison with all other Hymenopterans analyzed. The most significant gene loss events included genes in families of olfactory/odorant receptors, membrane proteins, and uncharacterized helix–turn–helix motifs across Hymenoptera. Gene gain events spanned families of transposases (e.g. harbinger), endonucleases involved in stress response in the *Bombus* lineages, and membrane transport proteins (e.g. carboxyl transferases).

## Discussion

Here, we present the first high-quality genome of the thelytokous parasitoid wasp, *D.coccinellae*, a species known for its uniquely solitary life history cycle, RNA viral-mutualism, and plasticity in parasitism across host coccinellid species. Our analyses indicate (1) ancient divergence of *D. coccinellae* from ancestral parasitoid wasps (∼136 MYA), (2) extensive gene duplications (∼10× more than *N.vitripennis*), (3) multiple independent evolutionary shifts to solitary behavior among Hymenoptera, (4) at least 2 independent shifts from ancestral arrhenotoky to thelytoky, and (5) accelerated evolution among several gene families along the *D. coccinellae* lineage.

The phylogeny of Braconid wasps is yet to be delineated using whole genomes, with most of the current work utilizing mitochondrial genes, and morphological information (Chen and van Achterberg 2019) to inform origins and divergence. Here, we utilize a phylogenomic approach to delineate an ancient divergence of Braconidae (here represented by *D. coccinellae*) from the common ancestor of other parasitoid wasps in the Jurassic–Cretaceous period (∼136 MYA). Braconid wasps are speciose, with a variety of endo- and ecto-parasitic life history strategies adapted to adult hosts among Coleoptera, Hemiptera, and Lepidoptera, with several independently evolved novel polydnavirus mutualisms (Herniou *et al.* 2013). Our work affirms the timeline proposed by Herniou *et al.* (2013) using polydnavirus genomes and lays the foundation for understanding models of viral-parasitoid wasp-host coevolution and diversification.

These timelines are also in line with previous work that establishes the timing of evolution of the 3 major modes of sex determination and reproduction across Hymenoptera: sexual reproduction, arrhenotokous parthenogenesis, and thelytokous parthenogenesis. Arrhenotokous parthenogenesis (arrhenotoky) has been determined to be the ancestral mode of sex determination and reproduction dating back to as far as 300 MYA, and presently remains the most prominent mode throughout the Hymenoptera order; arrhenotoky describes the process of sex determination in which diploid females develop from fertilized eggs and unfertilized eggs give rise to haploid males (Slobodchikoff and Daly 1971; Beukeboom *et al.* 2007; Heimpel and De Boer 2008). Thelytokous parthenogenesis (thelytoky) on the other hand is a convergently derived mode of sex determination and reproduction in which diploid female wasps are born from unfertilized egg clones (Slobodhcikoff and Daly 1971; Beukeboom *et al.* 2007; Heimpel and De Boer 2008; Kuhn *et al.* 2020).

Similarly, among Ichneumonoid parasitoid wasps, it is established that this apocritan superfamily consists of 2 main subfamilies: the Braconidae and Ichneumonidae sister clades (Belshaw and Quicke 2002; Quicke *et al.* 2020) with differential parasitism modes. Across these 2 sister branches, the different host parasitism strategies that parasitoid wasps employ center around how they exploit various developmental stages of their host. The first host exploitation strategy, idiobiosis, describes parasitoids that oviposit into immobilized hosts with paused development during the larval parasitoid’s growth, such as host eggs or cocooned juveniles; contrastingly, koinobiosis, describes parasitoids that oviposit into adult or larval hosts that continue to eat and develop further throughout larval parasitoid growth (Jervis and Ferns 2011; Harvey and Malcicka 2016). Generally, it has been determined that the host resource exploitation strategy employed most often by the Ichneumonidae subfamily tends to favor idiobiont ectoparasitoids, while the Braconidae subfamily often exploits their hosts through a koinobiont endoparasitoid strategy (Gauld 1988; Quicke and van Achterberg 1990). Between the Braconidae and Ichneumonidae sister subfamilies, ancestral members of both branches similarly externally exploit their hosts juvenile/immature stages (idiobiont ectoparasitoid), then go on to radiate across a range of different hosts over time (Gauld 1988). Our study therefore also affirms this timeline of adaptive evolution to host-parasitism.

**Fig. 2.**
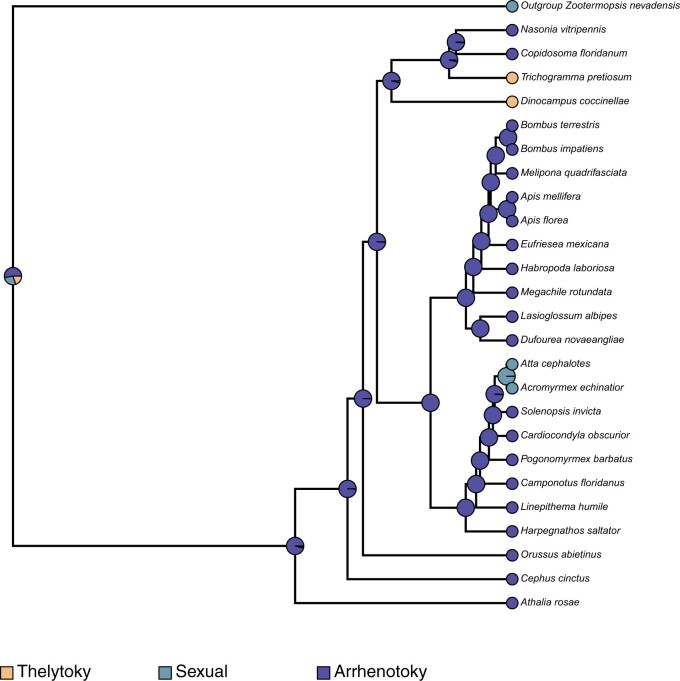
Ancestral state reconstruction using the stochastic character mapping method of Huelsenbeck *et al.* (2003), as implemented in phytools (Revell 2012), mapping the evolution of mode of parthenogenesis across all hymenopterans, in comparison with the outgroup, *Z. nevadensis.* The pies at internal and external nodes represent the posterior probability distribution of 1 of 3 possible states: (1) Thelytoky, (2) Arrhenotoky, and (3) Sexual reproduction.

**Fig. 3.**
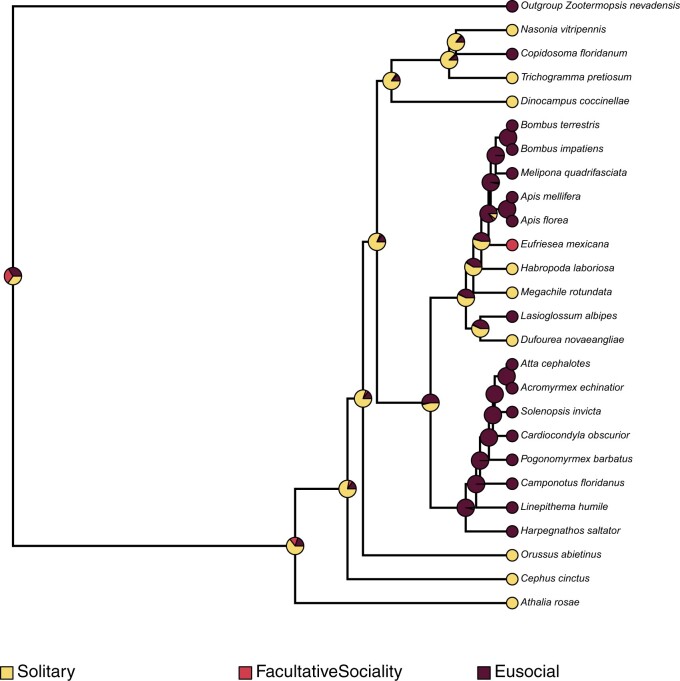
Ancestral state reconstruction using the stochastic character mapping method of Huelsenbeck *et al.* (2003), as implemented in phytools (Revell 2012), mapping the evolution of sociality across all hymenopterans, in comparison with the outgroup, *Z. nevadensis.* The pies at internal and external nodes represent the posterior probability distribution of 1 of 3 possible states: (1) Solitary, (2) Eusociality, and (3) Facultative sociality.

Contemporary analysis into the origins of thelytokous parthenogeny in Hymenoptera points to this reproductive strategy having convergently evolved from an ancestral arrhenotokous haplo-diploid state (Kuhn *et al.* 2020), with the conditional utilization of sexual reproduction to “restore” genetic diversity. However, the high morphological variability of *D. coccinellae*, despite the presence of a clonal genome, may suggest that the restoration of genetic diversity is unnecessary given their immense ability to change with their environment (Vansant *et al.* 2019). Ancestral state reconstruction in this study points to at least 2 independent evolutionary events leading to thelytoky among parasitoid wasps (Fig. 2), which also interestingly coincides with the evolution of solitary behavior in *D. coccinellae* and *T. pretiosum* (Fig. 3). Solitary behavior and thelytoky can be seen as complimentary behaviors as the absence of mates encourages asexual reproduction.

The evolution of eusociality is just one of many major transitions on Earth (Woodard *et al.* 2011). The transition to eusocial from solitary has occurred many times, mostly in insects, and only in a small number of lineages (Woodard *et al.* 2011). The evolution of eusociality is quite interesting since it requires a balance between cooperation and conflict with a preferential shift toward cooperation since this would be the only favorable outcome for fitness (Woodard *et al.* 2011). The selective pressures for individual success, such as in the case of the Braconid wasp *D.coccinellae*, require that the amount of energy put into the offspring outweighs the costs of forgoing reproduction to care for the offspring of others, as in the case of the *A.mellifera* where the well-being of the hive is one of the top priorities (Woodard *et al.* 2011).

Evidence for the influence of environmental factors suggests that relatedness and kinship may also play an important role in the development of eusociality (Hughes *et al.* 2008). However, there is also evidence for sociality determination through environmental factors (Soucy and Danforth 2002). Recent research of Hymenopteran chemoreceptors and their vast differentiation and specialization among different species have shown to play a major role in the emergence and development of eusocial behavior (Ferguson *et al.* 2021). Chemoreceptor genes and their frequencies are highly variable among Hymenopteran species, generally occurring in large expansions of eusocial species, but are also known to have lineage-specific patterns of losing or gaining genes due to tandem repeat events that result in unique clusters of chemoreceptor genes (Ferguson *et al.* 2021). Our genome-wide analyses of diversification and gene gain/loss events find consistent gene loss and gain among chemoreceptor and viral-coevolution genes along the *D. coccinellae* lineage. Further work utilizing gene-expression analyses is required to establish the functional significance of these genes among Hymenoptera.

## Data availability

The assembled genome has been deposited with NCBI and can be accessed via http://www.ncbi.nlm.nih.gov/bioproject/744197

All code, ab initio, homology-mediated gene predictions, and analyses scripts can be accessed at the corresponding author’s GitHub page: www.github.com/arunsethuraman/dcoccinellae

All annotation tracks and other relevant data files can be accessed at https://usegalaxy.org/u/rykamae/h/dcoccinellaegenome

Supplemental material is available at *G3* online.

## Supplementary Material

jkac001_Supplementary_Data
