# Supplementary material for: Genome of the parasitoid wasp Dinocampus coccinellae reveals extensive duplications, accelerated evolution, and independent origins of thelytokous parthenogeny and solitary behavior
Source: G3 (Bethesda). 2022 Jan 6;12(3):jkac001. doi: 10.1093/g3journal/jkac001 (PMC8896016; doi:10.1093/g3journal/jkac001)
Supplement: jkac001_Supplementary_Data [file jkac001_supplementary_data.docx]

**Supplementary Material to Sethuraman et al., 2021**

<Placeholder for SupplementaryTable1.xlsx>

Table 1 – Number of gene loss/gain events, variances, significance estimated across all Hymenopteran genomes analyzed in this study, along with hyperlinks to significantly gained/lost gene families. Please see file SupplementaryTable1.xlsx.

Table 2: Assembly statistics for the final Dovetail HiRise assembly of *D. coccinellae*

|  | **Number of elements** | **Length occupied (bp)** | **Percentage of sequence (%)** |
| --- | --- | --- | --- |
| **Retroelements** | 1024 | 1237966 | 0.68 |
| SINEs | 117 | 8236 | 0 |
| Penelope | 0 | 0 | 0 |
| LINEs | 154 | 266465 | 0.15 |
| CRE/SLACS | 0 | 0 | 0 |
| L2/CR1/Rex | 63 | 86798 | 0.05 |
| R1/LOA/Jockey | 0 | 0 | 0 |
| R2/R4/NeSL | 91 | 179667 | 0.1 |
| RTE/Bov-B | 0 | 0 | 0 |
| L1/CIN4 | 0 | 0 | 0 |
| LTR elements | 753 | 963265 | 0.53 |
| BEL/Pao | 495 | 395574 | 0.22 |
| Ty1/Copia | 0 | 0 | 0 |
| Gypsy/DIRS1 | 242 | 556885 | 0.31 |
| Retroviral | 0 | 0 | 0 |
| **DNA transposons** | 2651 | 1060863 | 0.58 |
| hobo-Activator | 78 | 76437 | 0.04 |
| Tc1-IS630-Pogo | 589 | 254058 | 0.14 |
| En-Spm | 0 | 0 | 0 |
| MuDR-IS905 | 0 | 0 | 0 |
| PiggyBac | 60 | 14814 | 0.01 |
| Tourist/Harbinger | 0 | 0 | 0 |
| Other (Mirage, Transib, P-element) | 0 | 0 | 0 |
| **Rolling-circles** | 554 | 409389 | 0.22 |
| **Unclassified** | 42684 | 13578880 | 7.46 |
| **Total interspersed repeats** |  | 15877709 | 8.72 |
| **Small RNA** | 1353 | 1415278 | 0.78 |
| **Satellites** | 0 | 0 | 0 |
| **Simple repeats** | 262267 | 11015885 | 6.05 |
| **Low complexity** | 51812 | 2934992 | 1.61 |

Table 3: Assessment of retroelements, transposable elements, and other repeats across the *D. coccinellae* HiRise assembly using RepeatModeler v.2.0.1 and RepeatMasker v.4.1.2-p1 (Smit et al., 2019), with further annotations from RepeatMasker obtained as a GFF3 track that can be visualized on the genome annotation using JBrowse.


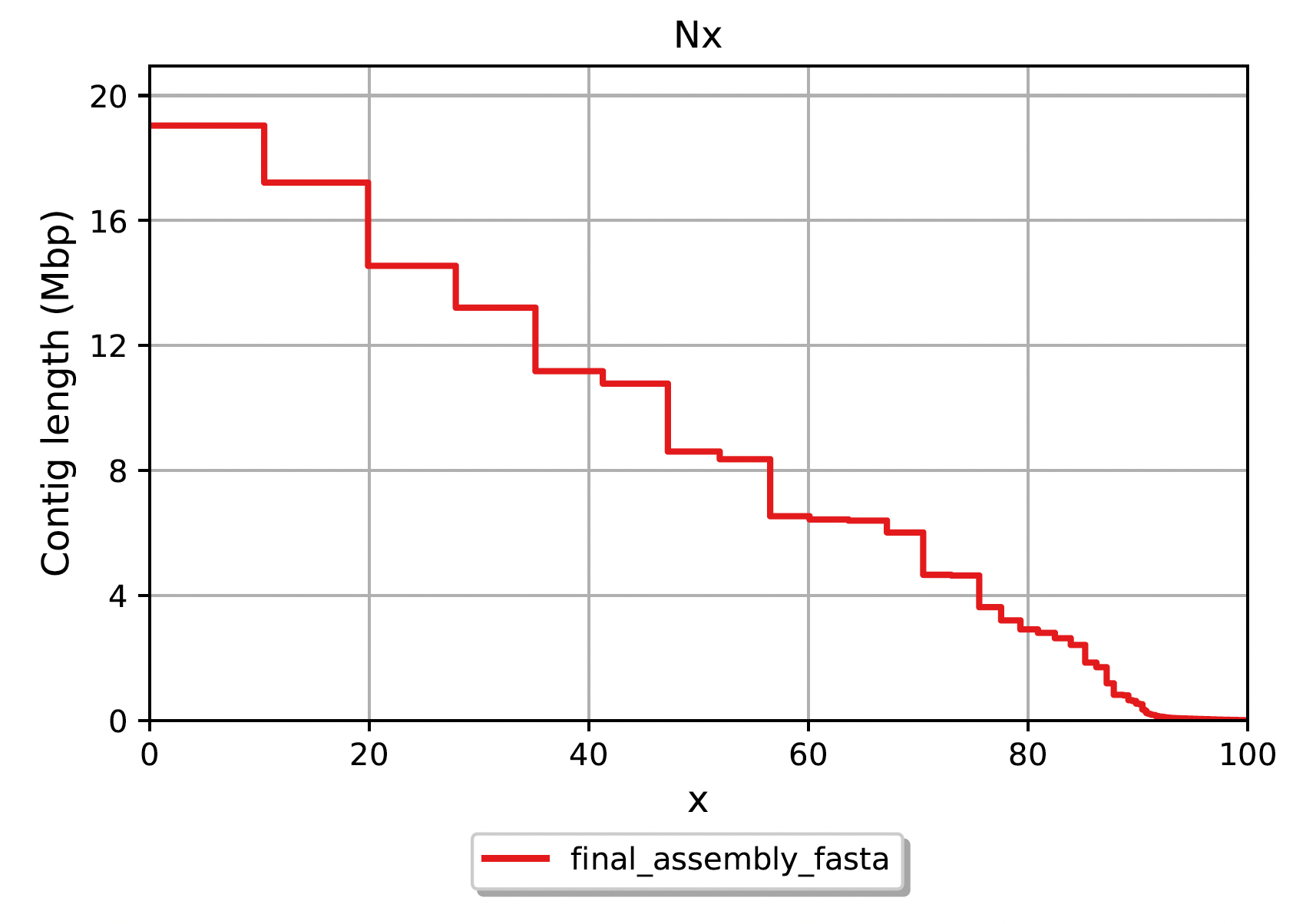


Fig. 1 Contig length distribution of the final Dovetail HiRise assembly for *D. coccinellae*


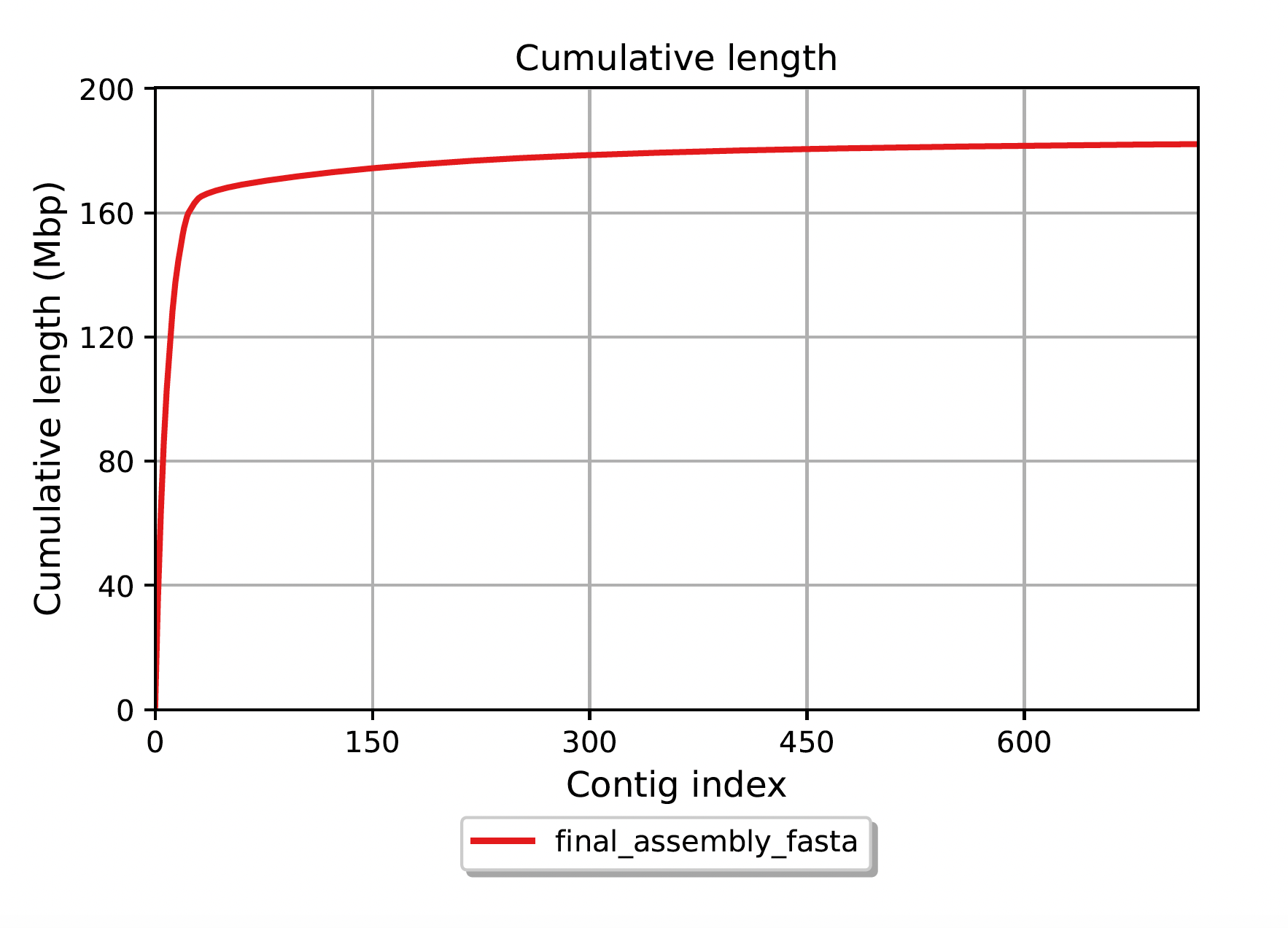


Figure 2: Cumulative length distribution of all contigs from the final Dovetail HiRise assembly for *D. coccinellae*


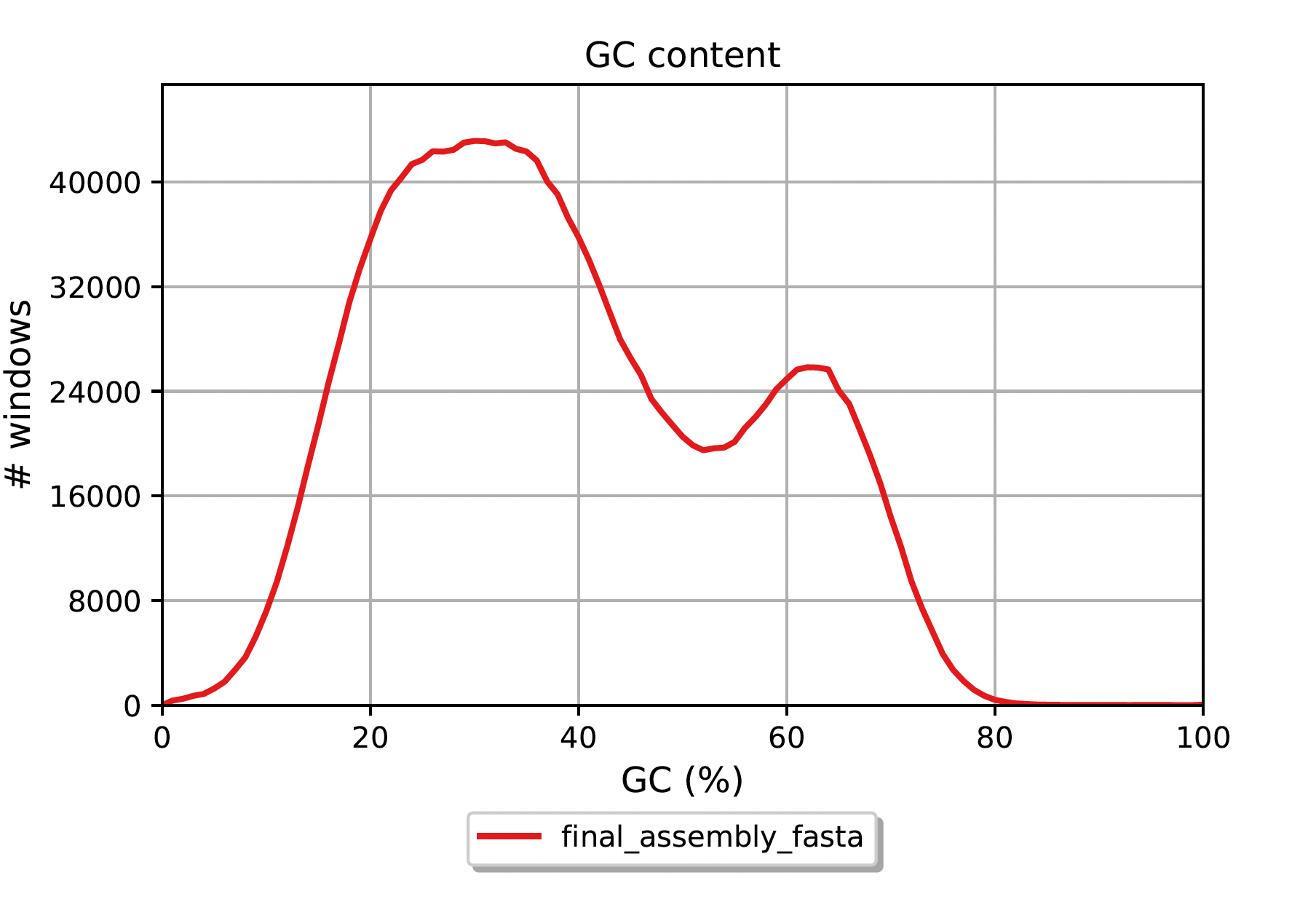


Fig. 3 GC-content distribution of the final Dovetail HiRise assembly of *D. coccinellae*
